# Response of Carbon Emissions and the Bacterial Community to Freeze–Thaw Cycles in a Permafrost-Affected Forest–Wetland Ecotone in Northeast China

**DOI:** 10.3390/microorganisms10101950

**Published:** 2022-09-30

**Authors:** Chao Liu, Xingfeng Dong, Xiaodong Wu, Dalong Ma, Yufei Wu, Haoran Man, Miao Li, Shuying Zang

**Affiliations:** 1 Heilongjiang Province Key Laboratory of Geographical Environment Monitoring and Spatial Information Service in Cold Regions, Harbin Normal University, Harbin 150025, China; 2 Heilongjiang Province Collaborative Innovation Center of Cold Region Ecological Safety, Harbin 150025, China; 3 Cryosphere Research Station on the Qinghai-Tibet Plateau, State Key Laboratory of Cryospheric Science, Northwest Institute of Eco-Environment and Resources, Chinese Academy of Sciences, Lanzhou 730000, China

**Keywords:** climate warming, permafrost degradation, freeze–thaw cycle, methane, carbon dioxide, bacterial community

## Abstract

Climate warming can affect freeze–thaw cycle (FTCs) patterns in northern high-latitude regions and may affect permafrost carbon emissions. The response of carbon release and microbial communities to FTCs has not been well characterized. Here, we conducted laboratory incubation experiments to investigate the relationships among carbon emissions, bacterial community, and soil variables in a permafrost-affected forest–wetland ecotone in Northeast China. The emission rates of CO_2_ and CH_4_ increased during the FTCs. FTC amplitude, FTC frequency, and patch type had significant effects on carbon emissions. FTCs increased the contents of soil DOC, NH_4_^+^-N, and NO_3_^−^-N but reduced bacterial alpha diversity. CO_2_ emissions were mainly affected by bacterial alpha diversity and composition, and the inorganic nitrogen content was the important factor affecting CH_4_ emissions. Our findings indicated that FTCs could significantly regulate CO_2_ and CH_4_ emissions by reducing bacterial community diversity and increasing the concentration of available soil substrates. Our findings shed new light on the microorganism-substrate mechanisms regulating the response patterns of the soil carbon cycle to FTCs in permafrost regions.

## 1. Introduction

The global air temperature is expected to increase by more than 1.5–2 °C in this century if no effective emission reduction measures are taken [1]. Under a warming climate, the carbon cycle in permafrost regions will make a major contribution to the global carbon budget due to its large amount of stored carbon and the high sensitivity of carbon release to temperature [2,3,4]. Permafrost degradation, including a deeper active layer, and reduction of permafrost areas have been widely observed in northern high latitudes [5,6,7], and this process can promote the microbial utilization of soil organic matter that has accumulated over the past thousands of years [8,9].

Climate warming can increase soil temperature and thaw permafrost, which can increase microbial activities and promote the decomposition of organic matter; it can also alter soil freeze–thaw cycle (FTCs) patterns. FTCs play a vital role in the soil biogeochemical process [10,11] via several mechanisms. First, phase changes of soil water in the freezing phase can destroy soil aggregate structure and promote the release of nutrients from soil lattices and colloids [12]. The melting of ice can increase the liquid water content of the soil, which can benefit the growth of anaerobic microorganisms [13]. Second, low temperature during the freezing stage can lead to the death of some microorganisms and plant roots. The nutrients and carbon substrate released from this organic matter can be used by survivors in the thawing stage to further enhance soil respiration [14,15]. Third, greenhouse gases produced during the freezing period remain in soil due to a physical barrier [16], and these gases can be released after the melting of ice [17]. Therefore, FTCs have a strong effect on CH_4_ and CO_2_ emissions, and peak values usually occur during the initial phase of spring and autumn FTCs [18]. Although the mechanisms and processes underlying the effects of FTCs on greenhouse gases have been studied, the quantitative effects of FTCs on carbon release have not yet been characterized. This knowledge gap can introduce significant uncertainty in future predictions of the permafrost carbon cycle.

In northern permafrost regions, climate warming rapidly leads to the degradation of permafrost [19], and this will alter the soil hydrothermal process and result in changes in vegetation composition and community succession [20,21]. Vegetation type can also affect soil carbon emissions [22,23]. Therefore, the ecological ecotone also requires consideration in studies of permafrost carbon release given that soil moisture levels, thermal regimes, and microhabitat types vary among ecological ecotones [24]. However, the response of soil carbon emissions and the bacterial community to the FTCs in different ecotones of permafrost regions are rarely known.

The DaXing’an Mountains, located on the southern boundary of the Eurasian permafrost zone, have experienced rapid warming over the past 100 years [25]. This area provides an excellent opportunity to examine the effects of FTCs on ecotones because it is a high-temperature permafrost region. Here, we collected soil samples from forest and wetland patches to investigate the effects of FTCs on the release rates of greenhouse gases and the factors driving them using laboratory experiments. The main goals of this study were to (1) determine the effect of FTC amplitude and frequency on soil carbon emissions and the bacterial community in different forest–wetland ecotones and (2) investigate the mechanisms underlying the regulation of the microbial community and substrate availability for CO_2_ and CH_4_ emissions under FTCs. We hypothesized that FTCs and ecotone type have a strong effect on soil carbon emissions and bacterial community structure, and FTCs might regulate CO_2_ and CH_4_ emissions by altering bacterial community diversity and increasing available soil substrates.

## 2. Materials and Methods

### 2.1. Site Description

**Figure 1:**
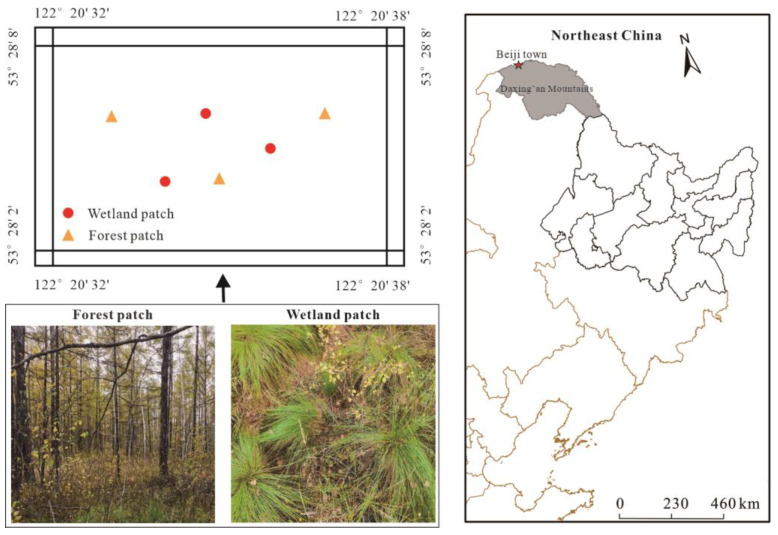
Sampling sites of forest–wetland ecotone in Northeast China.

This study was performed in a permafrost-affected forest–wetland ecotone in Beiji town, Daxing’an Mountains, Northeast China (Figure 1). The area experiences a typical cold temperate continental monsoon climate with an annual mean air temperature and precipitation of −2.19 °C and 549.9 mm, respectively [26]. The depth of the active layer is approximately 90–100 cm and 125–135 cm in wetland and forest patches, respectively, and measurements of the active layer were taken manually using a stainless steel drill in September 2020. The multiple diurnal FTCs begin in early May, and the spring FTC period lasts approximately two weeks; the autumn FTC period lasts approximately a week in mid-October [26]. The dominant species of the forest patches are *Larix gmelina* Ruprecht, *Ledum palustre* Linn., *Betula ovalifolia* Ruprecht, and *Vaccinium uliginosum* Linn., and *Eriophorum vaginatum* Linn. mainly occurs in wetland patches.

### 2.2. Soil Sampling and Experimental Design

In early September 2020, three *Larix gmelina* Ruprecht forest patches and *Eriophorum vaginatum* Linn. wetland patches in the forest–wetland ecotone were selected for sampling. Three soil cores (0–10 cm) were collected with a soil auger after removing the litter and aboveground vegetation in each patch [27,28]. The samples were divided into three subsamples. One subsample was used for bacterial analysis, the second subsample was sieved by a 4 mm mesh filter for incubation experiments and used for measurements of the content of soil dissolved organic carbon (DOC), ammonium nitrogen (NH_4_^+^-N), and nitrate nitrogen (NO_3_^−^-N) [29]; the rest of the soils were sieved through a 0.25 mm mesh filter after being air-dried to determine soil pH.

Fresh soil (equivalent to 20 g of dry soil) with field moisture levels was incubated in 500 mL glass bottles. The samples were preincubated at 5 °C for 5 days before the FTC experiment to restore the normal activity of soil microorganisms [30,31,32]. The soil was frozen for 24 h and thawed for 24 h, which corresponded to a single FTC. The two FTC amplitudes were −5–5 °C and −10–10 °C, respectively [33]. After preincubation (CK) and the 1st [32,33], 3rd (close to the shortest FTC duration of 10 cm soil depth, 5 days), and 9th FTCs (close to the longest FTC duration of 10 cm soil depth, 17 days) [26], 10 mL gas samples were collected with a syringe with a three-way valve, and were analyzed using a gas chromatograph (Agilent 7890B, USA) (Table 1). Emission rates of CO_2_ and CH_4_ were calculated according to Lang et al. [34].

The soil carbon and nitrogen contents were measured after preincubation and the 1st, 3rd, and 9th FTCs. The DOC content was extracted by mixing soil samples with distilled water, shaking for 30 min, then centrifuging at 8000 rpm for 20 min [35] and supernatant was filtered by 0.45 μm filter membrane and then determined by TOC analyzer (Multi N/C 2100 analyzer Analytik Jena, Germany). Extraction of the content of soil NH_4_^+^-N and NO_3_^−^-N was performed using 2 mol L^−1^ KCl solution and shaking for 1 h [36], and was measured with a continuous flowing analyzer (SAN++ Skalar, Netherlands). Measurement of soil pH used a pH meter (PHS-25, Shanghai, China) at 1:10 soil water ratio [37]. Soil moisture content was determined by the weighting method after drying at 105 °C for 12 h [38]. In this study, the soil water content and pH of the forest patches were 23.38 ± 0.81% and 5.04 ± 0.04, respectively; the soil water content and pH of the wetland patches were 84.41 ± 5.36% and 4.84 ± 0.01, respectively.

### 2.3. Soil Microbial Analysis

Soil bacterial community samples were taken after preincubation and the 1st, 3rd, and 9th FTCs. An E.Z.N.A.^®^ soil DNA kit (Omega Bio-tek, Norcross, GA, USA) was used for extracting genomic DNA of microbial communities. DNA quality was estimated using 1% agarose gel electrophoresis, and measured by NanoDrop 2000 spectrophotometer (Thermo Scientific, Waltham, MA, USA). Amplification of 16S rRNA gene occurred by the primer pairs 515F (5′-GTGCCAGCMGCCGCGG-3′) and 806R (5′-GGACTACHVGGTWTCTAAT-3′). The details of thermal cycling were pre-denaturation for 3 min at 95 °C, 27 cycles (30 s at 95 °C, 30 s at 55 °C, and 45 s at 72 °C), and ultimately extension at 72 °C for 10 min. Each sample had three replicates. An AxyPrep DNA Gel Extraction Kit was used for the purification of PCR products (Axygen Biosciences, Union City, CA, USA), quantification for Quantus™ Fluorometer (Promega, Madison, WI, USA), and sequencing was performed on Illumina MiSeq PE300 platform (Illumina, San Diego, CA, USA).

The quality of the original sequences was controlled using Fastp software [39] spliced with Flash software [40]; sequences were clustered according to 97% similarity using UPARSE software [41], and chimeras were removed [41,42]. Each sequence was annotated using RDP classifier [43], the Silva 16S rRNA database (Silva v138) was compared, and the comparison threshold was set to 70%. The OTU table was rarefied at the lowest sequencing depth of the experimental samples. All the raw sequences have been deposited in the NCBI database under accession number PRJNA872837.

### 2.4. Data Analysis

SPSS 20.0 was used for statistical analysis. Differences in the soil carbon content, nitrogen content, and carbon emissions among experimental treatments or patch types were evaluated using independent samples *t*-tests [44]. Three-way ANOVA was performed to evaluate the effect of FTC amplitude, FTC frequency, patch type, and interactions on soil carbon content, nitrogen content, and carbon emissions [45]. Stepwise regression analysis was performed to analyze the main controlling factors of soil carbon emissions [46]. Redundancy analysis (RDA) used CANOCO 5.0 to clarify the relationship between soil substrates and bacterial phyla [47]. Non-metric multidimensional scaling (NMDS) and partial least squares discriminant analysis (PLS-DA) were performed using the Majorbio cloud platform to clarify the feedback of bacterial community structure to FTCs [48,49,50]. Student’s *t*-tests were carried out to determine differences in alpha diversity among treatments [51]. Other figures were drawn in Origin 2022b.

## 3. Results

### 3.1. Soil Carbon Emissions of Forest and Wetland Patches after FTCs

**Figure 2:**
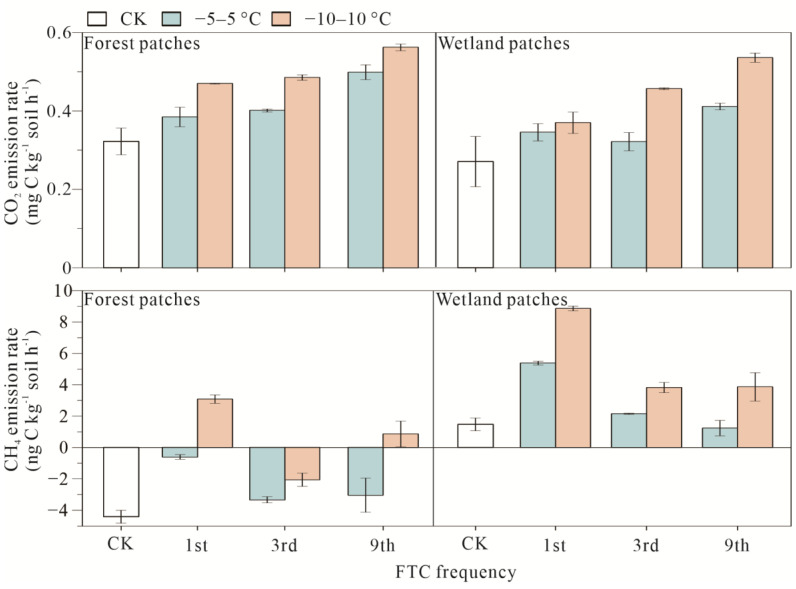
CO_2_ and CH_4_ emission rates of forest and wetland patches under different FTCs.

Compared with the preincubation period, higher CO_2_ and CH_4_ emissions were found during FTCs, and this effect was more pronounced in the −10–10 °C treatment (*p* < 0.05). FTC frequency increased the CO_2_ emission rates and decreased the CH_4_ emission rates in both the forest patches and wetland patches (Figure 2). The forest patches had a higher CO_2_ emission rate, and the wetland patches had a higher CH_4_ emission rate. FTC amplitude, FTC frequency, and patch type significantly affected CO_2_ and CH_4_ emissions (*p* < 0.05) (Table S1).

### 3.2. Soil Bacterial Community Composition and Diversity after FTCs

**Figure 3:**
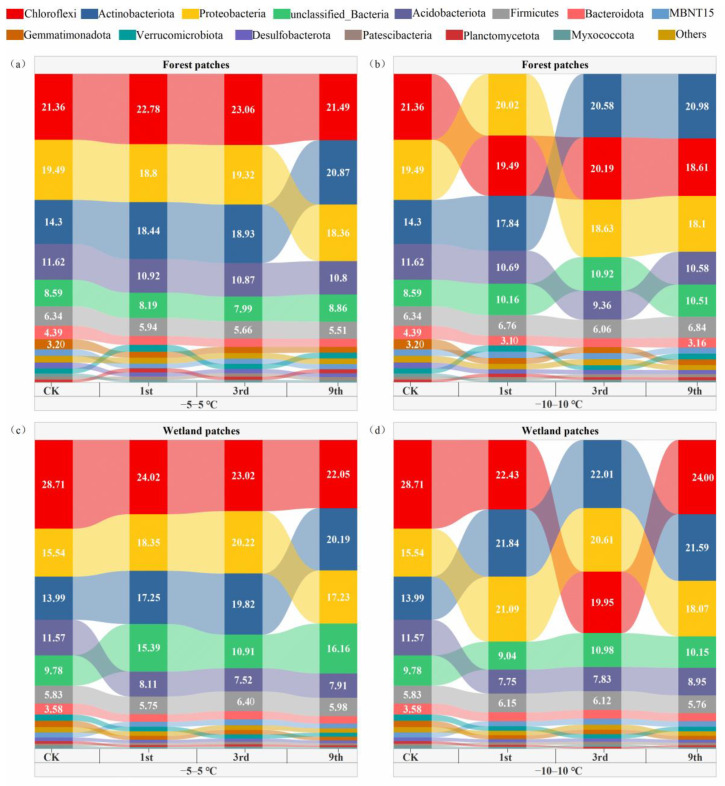
Relative abundance of soil bacterial phyla after FTCs. (**a**) forest patches, −5–5 °C FTC treatment; (**b**) forest patches, −10–10 °C FTC treatment; (**c**) wetland patches, −5–5 °C FTC treatment; (**d**) wetland patches, −10–10 °C FTC treatment. Values in column represent the relative abundance of bacteria on phylum level.

**Figure 4:**
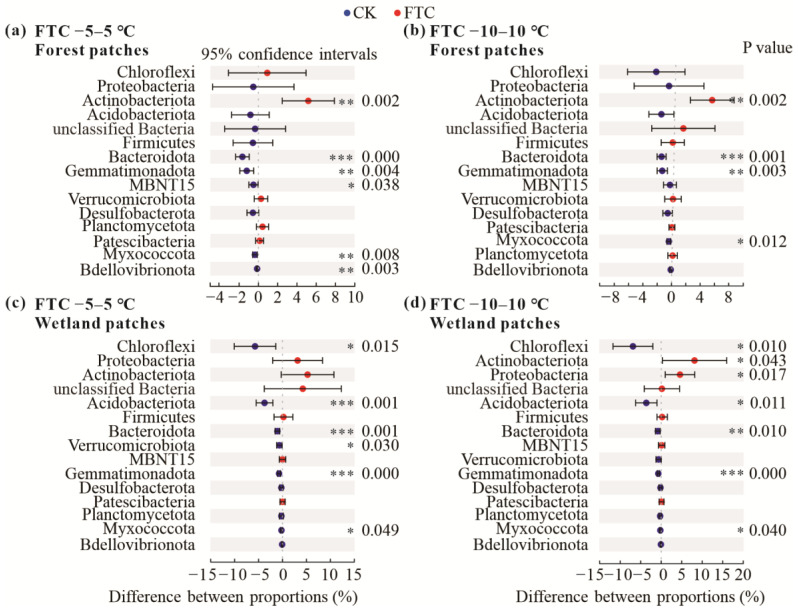
Differences in relative abundance of phyla (top 15) between preincubation and FTCs treatments (average of the 1st, 3rd, and 9th FTCs). (**a**) forest patches, −5–5 °C FTC treatment; (**b**) forest patches, −10–10 °C FTC treatment; (**c**) wetland patches, −5–5 °C FTC treatment; (**d**) wetland patches, −10–10 °C FTC treatment. Data are shown as the difference in proportions. * indicates *p* < 0.05; ** indicates *p* < 0.01; *** indicates *p* < 0.001.

Chloroflexi, Actinobacteriota, Proteobacteria, Acidobacteriota, and Firmicutes were the dominant phyla across all control samples and FTC treatments and accounted for more than 80% of all taxa, excluding unclassified bacteria (Figure 3). No significant differences were found in the relative abundance of bacterial phyla under treatments with different FTC frequencies. Therefore, we compared the average relative abundances of bacterial phyla in the treatments with the three FTCs with those of the preincubation samples. FTCs significantly increased the relative abundance of Actinobacteriota in forest and wetland patches under both FTC amplitudes (−5–5 °C and −10–10 °C). The relative abundances of Bacteroidota and Myxococcota decreased regardless of the FTC amplitude and patch type. Significant decreases were observed in a greater number of bacterial taxa in the wetland patches compared with the forest patches. Significant decreases in Chloroflexi and Acidobacteriota were only observed in the wetland patches (Figure 4).

**Figure 5:**
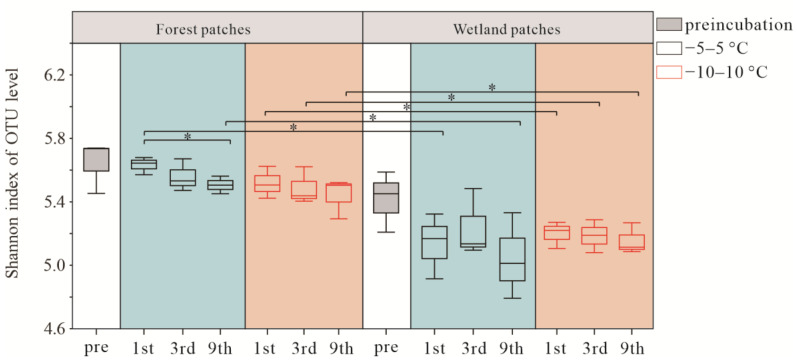
Bacterial alpha diversity under the different FTCs. * *p* < 0.05.

**Figure 6:**
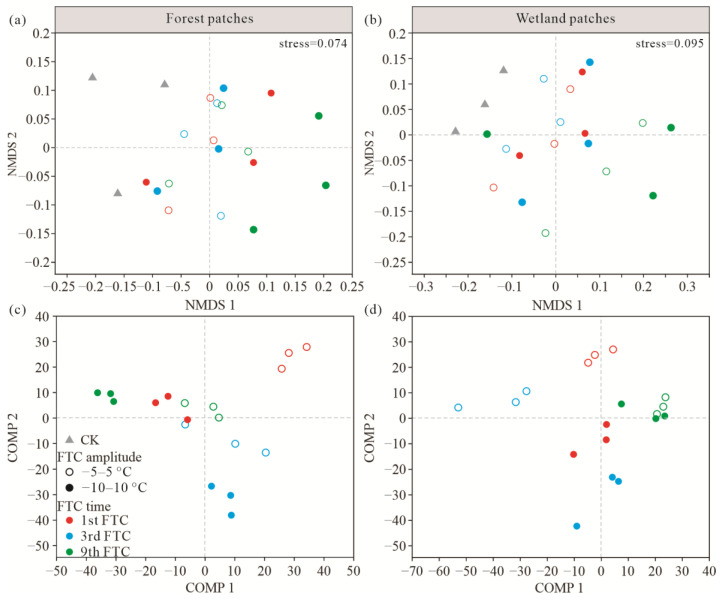
NMDS (**a**,**b**) and PLS-DA plots (**c**,**d**) revealing the effects of FTCs on the bacterial community. Hollow circles and solid circles indicate different FTC amplitudes. Hollow circles indicate FTCs at −5–5 °C; solid circles indicate FTCs at −10–10 °C.

Shannon index slightly decreased after FTCs, and the FTC amplitude had no significant effect on the Shannon index in both wetland and forest patches. The Shannon index in forest patches was significantly higher than that in wetland patches (Figure 5). The NMDS analysis revealed differences in the soil bacterial community between preincubation and FTC treatments (Figure 6a,b). To clarify the effects of FTC amplitude and frequency on bacterial communities, we used PLS-DA, by which random differences between groups were ignored and systematic differences between groups were highlighted (Figure 6c,d). Comp 1 revealed a contrast between the two FTC amplitudes in the wetland patches, suggesting that FTC amplitude in the wetland patches had significant effects on bacterial community structure (Figure 6d). Although comp 1 and comp 2 did not separate groups in the forest patches, obvious separation between groups was still observed (Figure 6c).

### 3.3. Soil Substrates of Forest and Wetland Patches after FTCs

**Figure 7:**
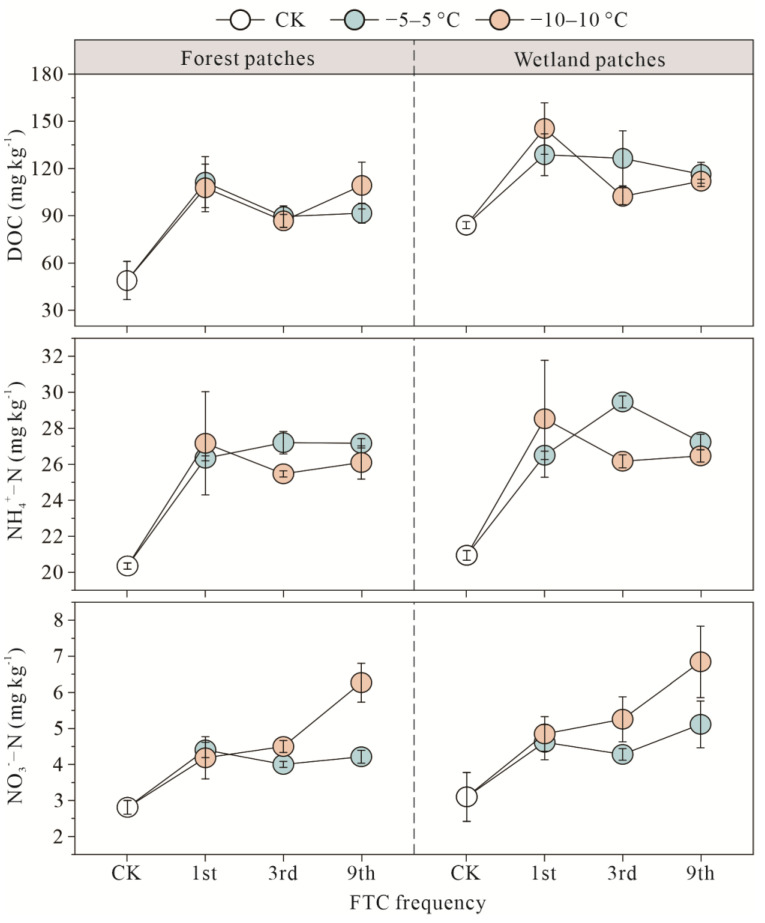
Effects of FTCs on soil DOC, NH_4_^+^-N, and NO_3_^−^-N contents.

The initial DOC content of the wetland patches and forest patches was 84.02 ± 2.26 mg kg^−1^ and 48.91 ± 12.09 mg kg^−^^1^, respectively. After various FTCs, the DOC content increased under the two FTC amplitudes (Figure 7). There was a significant difference in the average rate of increase in the DOC content between the forest patches (−5–5 °C: 99.36 ± 14.24%; −10–10 °C: 107.02 ± 14.86%) and wetland patches (−5–5 °C: 47.39 ± 4.57%; −10–10 °C: 42.73 ± 15.51%) (*p* < 0.05) (Table S2). FTCs had no significant effect on the NH_4_^+^-N content (Table S2). The percent increase in the content of NO_3_^−^-N was significantly higher under the −10–10 °C treatment (forest patches: 77.43 ± 23.08%; wetland patches: 82.37 ± 19.66%) than under the −5–5 °C treatment (forest patches: 49.71 ± 4.15%; wetland patches: 50.74 ± 7.81%).

### 3.4. Relationship among Soil Carbon Emissions, the Soil Bacterial Community, and Soil Substrates

**Figure 8:**
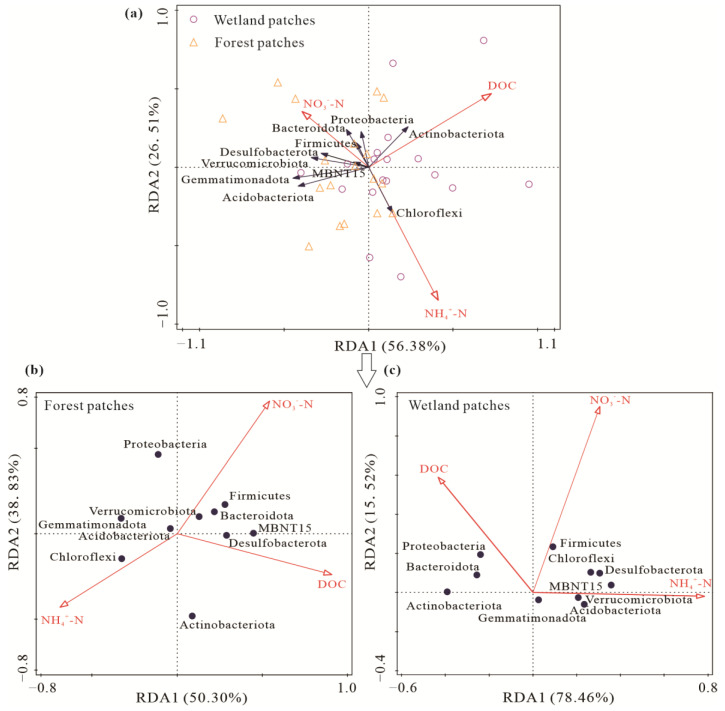
Redundancy analysis of soil bacterial phyla and the soil carbon and nitrogen contents in forest–wetland ecotone (**a**), forest patches (**b**) and wetland patches (**c**).

The two axes of the RDA explained 82.89% of the total variances between bacterial phyla and the soil carbon and nitrogen contents (Figure 8a). The DOC and NO_3_^−^-N contents were the most important factors affecting bacterial phyla, which explained 56.38% and 26.51% of the variances, respectively. The relative abundance of Actinobacteriota and Proteobacteria has a significantly positive relationship with soil DOC content, while it was significantly negative related with the NH_4_^+^-N content. The relative abundance of Chloroflexi showed a significantly positive correlation with soil NH_4_^+^-N content, while it was significantly negatively correlated with the NO_3_^−^-N content. The correlations between soil bacterial phyla and substrates were slightly different between two patch types (Figure 8b,c). In forest patches, the relative abundance of Proteobacteria was significantly negatively correlated with DOC content. In wetland patches, NH_4_^+^-N content was significantly positive correlated with Firmicutes and negatively correlated with Actinobacteriota. The correlations between Proteobacteria and DOC content as well as Chloroflexi and NO_3_^−^-N content were opposite for forest and wetland patches.

**Figure 9:**
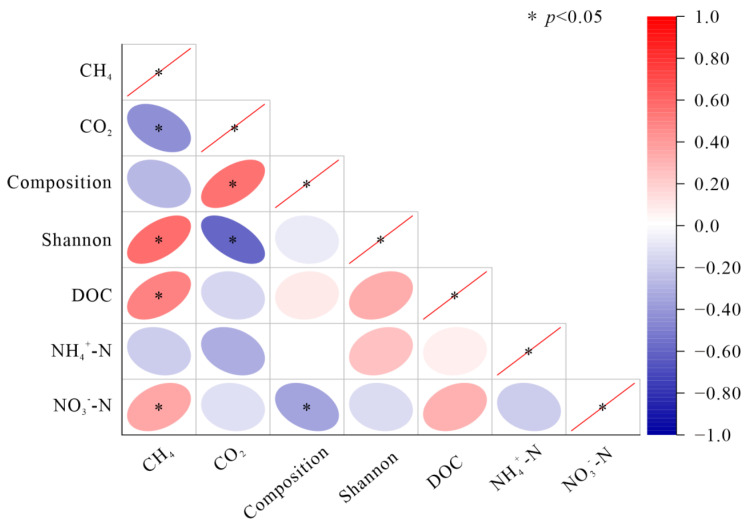
Pearson correlations among soil carbon emissions, bacterial diversity, and substrates after incubation. Red indicates positive correlations, and blue indicates negative correlations. * indicates significant correlations at the 0.05 level.

Correlation analysis showed that the CH_4_ emission rate was significantly positively correlated with soil DOC, NO_3_^−^-N contents, and Shannon index (Figure 9). CO_2_ emission rate had a significantly negative relationship with Shannon index, while it was positively correlated with bacterial composition (Figure 9). In addition, soil CH_4_ and CO_2_ emission rates showed the same correlation with substrates and bacterial diversity in different patches (Figure S1). To remove multicollinearity, stepwise multiple linear regression was used to find out the main biological and non-biological factors affecting CH_4_ and CO_2_ emissions in different patches (Table 2). Regardless of patch type, CO_2_ and CH_4_ emissions were affected by Proteobacteria. The main non-biological factors affecting the CH_4_ emission rate were NO_3_^−^-N in forest patches and the NH_4_^+^-N and NO_3_^−^-N content for wetland patches. Variation in CO_2_ emissions was largely explained by Chloroflexi, Proteobacteria, Actinobacteriota, MBNT5, and Gemmatimonadota for forest patches and by the Actinobacteriota, Proteobacteria, Acidobacteriota, Gemmatimonadota, and Desulfobacterota for wetland patches.

## 4. Discussion

### 4.1. Response of Carbon and Nitrogen to FTCs

In this study, FTCs increased the contents of DOC, NH_4_^+^-N, and NO_3_^−^-N both under the −5–5 °C and −10–10 °C treatments for the two patches. Previous findings also reported that DOC content significantly increased after FTCs in peatlands in Northeast China [52], pristine grassland soil of southern Edmonton [53], sub-arctic heath tundra mesocosms [54], and meadows in the Sanjiang Plain [55]. The FTC amplitude had no significant effects on DOC content, which could be due to the fact that DOC is easily mineralized by microorganisms [56]. In this study, the soil carbon emission increased with FTC amplitude, which might indicate a rapid consumption of DOC at the −10–10 °C treatment. The FTCs can significantly increase NH_4_^+^-N content [57], which is related to the conversion of nitrogen in soil [58]. During FTCs, the NO_3_^−^-N content increases when the production of nitrogen via mineralization and nitrification is higher than its consumption via denitrification [55]. With the increase of FTC frequency, the increase rate of carbon and nitrogen contents reduced, indicating that the first FTC already stimulated the maximum release of soil substrates [58,59]. Generally, FTCs increase the contents of carbon and nitrogen by killing microbial cells [60], which can release nutrients inside microbial cells into soil solution [61]. FTCs can also physically destroy soil aggregates and release nutrients [62,63]. The organic macromolecules will break down into smaller organic molecules when combined with soil aggregates, which further increases the DOC content [64].

### 4.2. Soil Bacterial Community Composition and Diversity Respond to FTCs

The response of the relative abundance of bacteria to FTCs varied among phyla. Some bacterial phyla are resistant to FTCs, and FTCs eventually result in decreases in less competitive phyla [65]. In this study, FTCs significantly increased the relative abundance of Actinobacteriota, which was consistent with previous studies [65,66,67,68]. This might be related to the filamentous and spore-forming properties of Actinobacteria, which can improve their ability to acquire resources during FTCs [69]. Männistö et al. [70] found that the relative abundances of Proteobacteria in arctic tundra significantly decreased after FTCs. In this study, FTCs decreased the abundance of Proteobacteria in the forest patches, which might stem from the fact that the increase in the abundance of Actinobacteriota was greater in the forest patches than in the wetland patches. Actinomycetes can produce secondary metabolites such as antibiotics under pressure, which allows them to outperform Proteobacteria [71]. Firmicutes include spore-forming groups such as Clostridia, which leads to its resistance to environmental interference [72]. The relative abundance of Firmicutes in both forest and wetland patches did not respond significantly to FTCs [70]. Gemmatimonadota prefer dry environments [73], and freezing may reduce the soil water supply and result in desiccation; however, the subsequent thawing process might induce rapid changes in the osmotic pressure balance inside and outside of cells, which can result in decreases in microbial activity [74]. Thus, the relative abundances of Gemmatimonadota decreased significantly after FTC treatment in Han et al. [66].

In this study, the relative abundance of Actinobacteriota and Proteobacteria has a significantly positive relationship with soil DOC content, which was agreed with Fierer et al. [75] and Schostag et al. [76]. The oligotrophic Acidobacteria significantly negatively correlated with soil DOC content [77]. Moreover, the relative abundance of Acidobacteria showed significant correlation with inorganic nitrogen content in different patches because Acidobacteria have multiple transport systems that promote ammonia, amino acids, and maintain growth by immobilizing inorganic nitrogen in the soil [78,79]. The correlations between bacterial phyla and substrates were slightly different between two patch types, which is reasonable because the original physico-chemical variables are different. For example, soil water contents greatly affected the changes in bacterial community composition [80].

Shannon index slightly decreased after FTCs, indicating that richness declined after FTCs. Changes in soil moisture, temperature, and substrate can directly or indirectly affect bacterial community structure [50,81,82]. In our study, the FTC amplitude and frequency had no significant effects on soil bacterial alpha diversity, but significant differences were observed between the two patches. These findings were consistent with the study of Ji et al. [83], showing that the bacterial alpha diversity of permafrost reflects adaptation to FTC stress. Generally, FTCs cause changes in the water phase, which changes the microbial niche and, thus, affects microbial diversity [84], and the significant differences in the soil water content between forest and wetland patches might explain the differences in soil bacterial alpha diversity between the two patches. NMDS and PLS-DA showed that both the amplitude and frequency of FTCs affected soil bacterial beta diversity, indicating that FTCs altered the structure of the soil bacterial community [85,86,87].

### 4.3. Response of Soil Carbon Emissions to FTCs and Its Relationship with the Soil Bacterial Community and Soil Substrates

FTCs increased the emission rates of CO_2_ and CH_4_, and this has been demonstrated in several previous studies [32,33,88,89,90]. The increases in greenhouse gas emissions might be related to the increases in soil carbon and nitrogen content caused by FTCs. Generally, FTCs destroy soil aggregates and microbial cells through a transition phase of soil moisture and low temperature, thereby releasing protected organic matter and enhancing the accessibility of substrates for microorganisms [91,92]. FTC amplitude, FTC frequency, and patch type significantly affected the CO_2_ and CH_4_ emission rates. FTC amplitude was significantly correlated with soil carbon emissions, which can be explained by the fact that high-intensity FTCs can supply more nutrients [93,94]. Although carbon emissions gradually decrease when readily available soil substrates are depleted [58], there were only nine FTCs in our experiment, which might be far from the number of FTCs that would be required to generate limitations in the amount of available substrate.

There were significant correlations among soil substrates, the bacterial community, and soil carbon emissions, and the stepwise regression analysis showed that the main factors affecting CO_2_ and CH_4_ emissions were bacterial community and inorganic nitrogen content. This result indicated that FTCs affect carbon emissions via soil substrates and the bacterial community. Dong et al. [95] found there were significantly positive relationships between CO_2_ emissions and most bacterial phyla abundances and diversity. Microbial community structure in the permafrost region is also a good predictor of CO_2_ emissions [96]. Actinobacteriota can perform refractory carbon degradation [97], and their genomes are thought to be rich in glycoside hydrolases that show good performance in cellulose, starch, and xylan degradation [98]. Proteobacteria are thought to have a positive correlation with the availability of carbon [76,99]. Significant negative relationships were observed between Gemmatimonadota and CO_2_ emissions [100,101]. Chloroflexi can affect CO_2_ emissions because the class Anaerolineae comprises anaerobic heterotrophic bacteria capable of decomposing carbohydrates and amino acids [102,103]. We also found that CH_4_ emissions were significantly correlated with NH_4_^+^-N and NO_3_^−^-N contents, which is consistent with the results of Jiang et al. [104] and Zhang et al. [105]. This finding likely stems from the ability of NO_3_^−^-N and NH_4_^+^-N to inhibit CH_4_ oxidation [106,107].

## 5. Conclusions

We examined the response of soil carbon emissions to FTCs in forest–wetland ecotone in a permafrost zone. Based on laboratory incubation experiments, we found that soil substrates as well as CO_2_ and CH_4_ emissions increased after the FTC treatment. FTCs did not change the dominant phyla taxa, but greatly affected the bacterial communities during different FTCs. FTC frequency, FTC amplitude, and patch type significantly affected CO_2_ and CH_4_ emissions. CH_4_ emission was affected by inorganic nitrogen content, and bacterial diversity and composition were the main factors affecting CO_2_ emissions. The relationship among carbon emissions, soil substrates, and bacterial community suggested that the accelerating effects of FTCs on CO_2_ and CH_4_ emissions were mainly related to the increasing microbial utilization of substrates, and FTC should be considered in the estimation of permafrost carbon emissions in a changing climate.

## Figures and Tables

**Table 1:** Details of the FTC simulation experiment.

Ecotone	Soil Water Content	FTC Amplitude	FTC Period	Sampling Time
Forest patches	Field water content (23.38 ± 0.81%)	−5–5 °C and −10–10 °C	2 days (the treated sample was frozen at −5 °C or −10 °C for 24 h and thawed at 5 °C or 10 °C for 24 h)	After preincubation (CK) and the 1st, 3rd, and 9th FTCs
Wetland patches	Field water content (84.41 ± 5.36%)			

**Table 2:** The biological and abiotic factors related to CO_2_ and CH_4_ emission rates in forest and wetland patches.

Patch type			Adjusted R^2^	F-Statistic	*p*	Significant Variables (*p* < 0.05)	Beta Coefficient
Forest patches	Biological factors	CH_4_	0.894	5.751	0.035	Proteobacteria	0.575
						Bacteroidota	1.145
		CO_2_	0.878	18.487	<0.001	Chloroflexi	−0.547
						Actinobacteriota	0.295
						Proteobacteria	−0.423
						MBNT15	0.646
						Gemmatimonadota	−0.608
	Non-biological factors	CH_4_	0.456	11.254	0.029	NO_3_^−^-N	0.536
		CO_2_	0.312	8.727	0.009	DOC	0.594
Wetland patches	Biological factors	CH_4_	0.316	13.617	0.04	Proteobacteria	1.689
						Firmicutes	−1.295
						Patescibacteria	−1.287
		CO_2_	0.729	8.643	0.01	Actinobacteriota	2.181
						Proteobacteria	−1.370
						Acidobacteriota	0.646
						Gemmatimonadota	0.672
						Desulfobacterota	0.518
	Non-biological factors	CH_4_	0.689	19.842	0.009	NH_4_^+^-N	−0.712
						NO_3_^−^-N	0.615
		CO_2_	-	-	-	None	-

Note: For the linear regressions, we standardized predictors (mean = 0, SD = 1) to permit the interpretation of coefficients as effect sizes.

## Data Availability

Data are available on request.

## Supplementary Materials

The following supporting information can be downloaded at: https://www.mdpi.com/article/10.3390/microorganisms10101950/s1. Table S1: Three-way factorial ANOVA of the effects of FTC amplitude, FTC frequency, patch type, and their interactions on CO_2_ and CH_4_ emission rates. Table S2: Three-way ANOVA for evaluating the effects of FTC amplitude, FTC frequency, patch type, and their interactions on DOC, NH_4_^+^-N, and NO_3_^−^-N contents. Figure S1: Pearson correlations among soil carbon emissions, bacterial diversity, and substrates in forest and wetland patches.

## Abbreviations

FTCs	freeze–thaw cycles
CO_2_	carbon dioxide
CH_4_	methane
DOC	dissolved organic carbon
NH_4_^+^-N	ammonium nitrogen
NO_3_^−^-N	nitrate nitrogen
RDA	redundancy analysis
NMDS	non-metric multidimensional scaling
PLS-DA	partial least squares discriminant analysis
