# Supplementary material for: Response of Carbon Emissions and the Bacterial Community to Freeze–Thaw Cycles in a Permafrost-Affected Forest–Wetland Ecotone in Northeast China
Source: Microorganisms. 2022 Sep 30;10(10):1950. doi: 10.3390/microorganisms10101950 (PMC9609725; doi:10.3390/microorganisms10101950)
Supplement: Supplementary file 1 [file microorganisms-10-01950-s001.zip › microorganisms-1907372-supplementary.pdf]

Table S1. Three-way factorial ANOVA of the effects of FTC amplitude, FTC frequency, patch type, and their interactions on CO_2 and CH_4 emission rates.

	**CO_2**		**CH_4**	
	F	***P***	***F***	***P***
FTC amplitude	142.336	<0.001	39.462	<0.001
FTC frequency	122.550	<0.001	79.613	<0.001
Patch type	173.475	<0.001	271.980	<0.001
FTC amplitude×FTC frequency	14.391	0.021	8.609	0.002
FTC frequency×Patch type	14.566	0.011	17.985	<0.001
FTC amplitude×Patch type	7.272	0.560	0.012	0.012
FTC amplitude×FTC frequency×Patch type	4.100	0.544	12.532	<0.001

Table S2. Three-way ANOVA for evaluating the effects of FTC amplitude, FTC frequency, patch type, and their interactions on DOC, NH_4^+-N, and NO_3^--N contents.

	**DOC**		**NH_4^+-N**		**NO_3^--N**	
	F	***P***	***F***	***P***	***F***	***P***
FTC amplitude	<0.001	0.990	0.770	0.389	9.199	0.006
FTC frequency	3.776	0.038	0.103	0.902	6.392	0.006
Patch type	11.038	0.003	1.171	0.290	3.797	0.063
FTC amplitude×FTC frequency	0.964	0.396	2.279	0.124	3.587	0.043
FTC frequency×Patch type	0.430	0.656	0.233	0.794	0.094	0.911
FTC amplitude×Patch type	0.319	0.577	<0.001	0.988	0.120	0.732
FTC amplitude×FTC frequency×Patch type	1.060	0.362	0.287	0.753	0.202	0.818

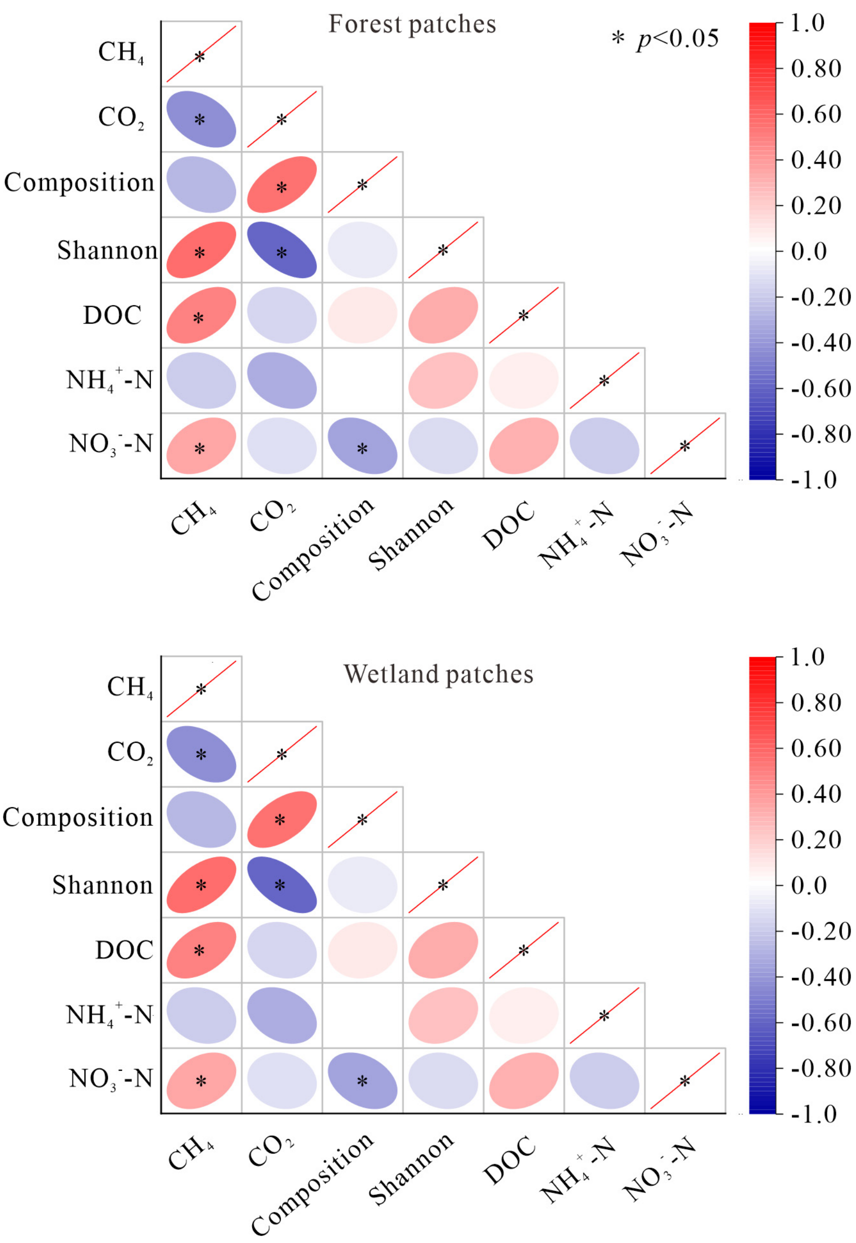

Figure S1. Pearson correlations among soil carbon emissions, bacterial diversity, and substrates in forest and wetland patches. Red indicates positive correlations, and blue indicates negative correlations. * indicated significant correlations at the 0.05 level.
